# Mosses of Gunung Senyum Recreational Forest, a tropical limestone forest in Pahang, Peninsular Malaysia

**DOI:** 10.3897/phytokeys.128.33860

**Published:** 2019-07-25

**Authors:** N. Norhazrina, N. Syazwana, M. Aisyah, H. Aznani, H. Maideen, M.S. Nizam

**Affiliations:** 1 Faculty of Science and Technology, Universiti Kebangsaan Malaysia, 43600 Bangi, Selangor, Malaysia Universiti Kebangsaan Malaysia Bangi Malaysia

**Keywords:** Mosses, limestone hill, Gunung Senyum Recreational Forest, Peninsular Malaysia

## Abstract

Gunung Senyum Recreational Forest harbours 59 species, two subspecies and five varieties of mosses in 32 genera and 16 families that had been identified from a total of 589 specimens collected from the area. These figures represent 11.8% out of the 558 taxa, 20.2% out of the 158 genera and 34.7% out of the 46 families of mosses reported for Peninsular Malaysia. The total also represents 14.9% of the 442 taxa, 24.0% of the 133 genera and 40.0% of the 40 families of mosses recorded in Pahang. The largest family of mosses found in this limestone forest is Calymperaceae followed by Fissidentaceae. There are two new records for Pahang, *Calymperespallidum* Mitt. and *Taxitheliumbinsteadii* Broth. & Dixon. The analysis of species similarities of mosses found in the study area with some other selected areas showed that Gunung Senyum Recreational Forest had a high percentage of species similarity with Perlis State Park at Wang Kelian, another limestone forest, at 38%. Corticol is the main habitat utilised by mosses in Gunung Senyum Recreational Forest with 47 taxa, followed by the lignicol and calcicol each with 35 and 26 taxa, respectively.

## Introduction

Forested limestone areas in Peninsular Malaysia are estimated at about 26,000 ha, mostly concentrated in the northern states and 50,000 ha in Sabah and Sarawak according to the World Wildlife Fund Malaysia (2018). More than 300 scattered limestone outcrops have been found in the Peninsular Malaysia which consist of limestone islands in the Langkawi archipelago, with major outcrops in Kelantan, Perlis, Kedah, Perak and northern Pahang.

Gunung Senyum Recreational Forest is one of the limestone forests in Peninsular Malaysia and is located in Jengka, Pahang (latitude 3°43.0683’N and longitude 102°26.0043’E). This recreational forest consists of several series of limestone hills, including Gunung Senyum and Gunung Jebak Puyuh. They are hills located northeast of Temerloh and north of Kampung Awah (Fontaine et al. 1988). In addition to limestone hills, this area also comprises lowland forest, especially in the area between Gunung Senyum and Gunung Jebak Puyuh. Gunung Senyum has 18 caves, while seven caves have been recorded at Gunung Jebak Puyuh. These caves originated from weathering activities where some of them are archaeological sites. Several studies in geological, human civilisation and rock life have been conducted in the areas of Jengka including Gunung Senyum and Gunung Jebak Puyuh by Jasin et al. (1995) and Fontaine et al. (1988).

The collection of plant specimens from limestone hills in Peninsular Malaysia started in August 1880 by Kunstler, a collector for Sir George King, who collected specimens in the limestone area of Gopeng, Perak. In addition, there are other collectors, namely Fox, Ridley, Kelsall and Wooldridge. As a result, about 4,500 plant specimens have been collected (Chin 1977).

The study of the limestone flora in most parts of Peninsular Malaysia was initiated by Henderson from 1923 to 1935, including Gunung Senyum and other limestone areas where about 745 plant taxa were recorded here. Also, Carr had collected plant specimens at Gunung Senyum from 1928 to 1930 but most specimens collected by him are orchids and ferns (Chin 1977). Then, Chin (1977) listed about 1216 plant taxa in a comprehensive study of limestone hills in Peninsular Malaysia consisting of pteridophytes, angiosperms and gymnosperms. He also listed Gunung Senyum as one of the largest distribution area of limestone hills in Peninsular Malaysia.

The first comprehensive study on limestone moss flora in Peninsular Malaysia was conducted by Mohamed (1987), in which about 21 limestone outcrops mainly in the northern half of the country were surveyed. He listed about 73 taxa in 40 genera and 18 families of mosses. After that, Damanhuri and Maideen (2001) recorded a total of 71 taxa in 34 genera and 18 families of mosses in Perlis State Park, Wang Kelian, Perlis. Yong et al. (2002) collected about 57 taxa in 28 genera and 13 families of mosses in Wang Mu Forest Reserve, Perlis State Park, Perlis. Later, Damanhuri et al. (2007) reported about 112 taxa in 45 genera and 19 families of mosses in Kenong Forest Park, Pahang. Lastly, Kiew et al. (2014) listed about 25 taxa in 14 genera and 11 families of mosses found in Gunung Kanthan, Perak.

Some studies in Gunung Senyum had been reported before by Chin (1977), but their collections did not cover mosses. Until now, the Gunung Senyum Recreation Forest had not been explored in terms of its moss flora. Therefore, this is the first study of moss conducted there. Also, from this study, a new record for Peninsular Malaysia has been made in this area, *Calymperespallidum* Mitt. (Ellis et al. 2018).

## Methods

**Figure 1.**
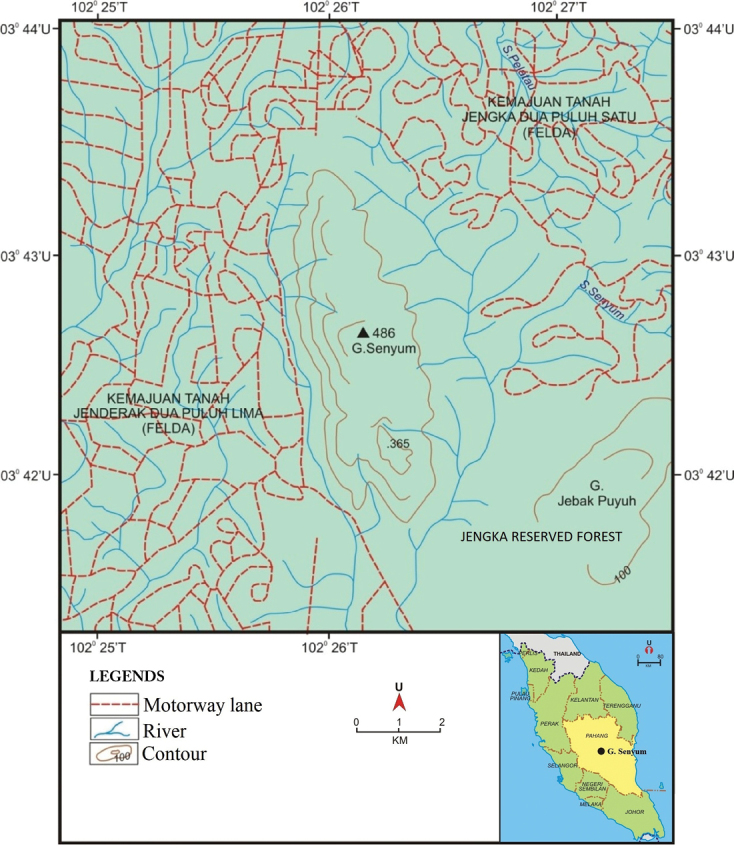
Map of Gunung Senyum Recreational Forest, Jengka Forest Reserve, Pahang.

This study is based on samples collected at Gunung Senyum Recreational Forest located in the Jengka Reserved Forest, Pahang (Figure 1). Collections were made along the trails in the Gunung Senyum Recreational Forest. All the specimens are curated and deposited in the Herbarium of Universiti Kebangsaan Malaysia (UKMB).

The information regarding collection numbers, altitudes of each sample collected, date and locality of each specimen collected in Gunung Senyum Recreational Forest are shown in Table 1. Various microhabitats of mosses such as tree trunks, buttresses, rotten logs, surfaces and crevices of rocks, soil and soil banks were carefully surveyed in order to obtain as many samples and species as possible.

**Table 1.** Collection information of moss specimens collected in Gunung Senyum Recereational Forest.

**Date**	**Altitude (m)**	**Specimen No.**	**Locality**
16/08/2009	85–170	1–90	The foot of Gunung Senyum
17/08/2009	75–485	91–264	Trails from the foot to the summit of Gunung Senyum
18/08/2009	95–l60	265–589	Trails to Gunung Jebak Puyuh and the surrounding areas

## Results and discussion

A total of 59 species, two subspecies and five varieties of mosses in 32 genera and 16 families was found in Gunung Senyum Recreational Forest, Pahang (Table 2 and Appendix 1). These numbers represent 11.8% of the 558 taxa, 20.2% of the 158 genera and 34.7% of the 46 families of mosses reported in Peninsular Malaysia. Based on the records of mosses found in Pahang, these figures represent 14.9% of the 442 taxa, 24.0% of the 133 genera and 40.0% of the 40 families of mosses in the state. Two species are new additions to the bryoflora of Pahang namely *Calymperespallidum* Mitt. and *Taxitheliumbinsteadii* Broth. & Dixon, where *C.pallidum* had been published as a new record for Peninsular Malaysia (Ellis et al. 2018). Meanwhile, a *Fissidens* species remains unidentified and requires further study to ascertain its true identity. This species will contribute to new findings for the genus in Peninsular Malaysia as for the time being there are about 28 taxa recorded in Peninsular Malaysia (Syazwana et al. 2018). The new discoveries of moss species in this area can also increase the bryoflora of Pahang in which the current record is about 442 taxa of mosses in 133 genera and 40 families. The total is higher compared to neighbour states such as Kelantan (299 taxa in 105 genera and 37 families) and Terengganu (253 taxa in 88 genera and 31 families). This shows that Pahang has the highest record of moss species compared to other states in the east coast region of Peninsular Malaysia.

**Table 2.** Summary of mosses found in Gunung Senyum Recreational Forest and its vicinity.

**No.**	**Families**	**Genera**	**Species & infraspecific taxa**
1	Bartramiaceae	1	1
2	Brachytheciaceae	1	1
3	Bryaceae	1	1
4	Calymperaceae	6	18 spp. + 2 subsp.
5	Fissidentaceae	1	8 spp. + 2 var.
6	Hypnaceae	2	6
7	Leucomiaceae	1	1
8	Meteoriaceae	1	1
9	Neckeraceae	4	6
10	Orthotrichaceae	1	1
11	Pilotrichaceae	1	1 var.
12	Plagiotheciaceae	1	1
13	Pottiaceae	3	3
14	Pylaisiadelphaceae	2	5 spp. + 1 var.
15	Sematophyllaceae	5	4 spp. + 1 var.
16	Thuidiaceae	1	2
	Total	32	59 spp., 2 subsp., 5 var.

Amongst the 15 families recorded, Calymperaceae has the highest number of members (20 taxa), followed by Fissidentaceae with nine taxa. Hypnaceae, Neckeraceae and Pylaisiadelphaceae are the third largest families, each represented by six taxa. This is followed by Sematophyllaceae with five taxa, Pottiaceae and Thuidiaceae with three and two taxa, respectively. The rest, Bartramiaceae, Brachytheciaceae, Bryaceae, Leucomiaceae, Meteoriaceae, Orthotrichaceae, Plagiotheciaceae and Pilotrichaceae have one taxon each (Table 2).

The largest genus found in this study is *Calymperes* with 11 taxa which belongs to the largest family (Calymperaceae) recorded here. *Fissidens* is the second largest with nine taxa. *Taxithelium* is the third largest with five taxa followed by *Vesicularia* and *Mitthyridium* with four and three taxa respectively. Genera represented by two taxa each are the *Ectropothecium*, *Leucophanes*, *Neckeropsis*, *Pelekium*, *Pinnatella*, and *Syrrhopodon*. The remainder, *Acanthorrhynchium*, *Acroporium*, *Arthrocormus*, *Barbula*, *Bryum*, *Caduciella*, *Callicostella*, *Circulifolium*, *Exostratum*, *Floribundaria*, *Hyophila*, *Isopterygium*, *Leucomium*, *Macromitrium*, *Meiothecium*, *Papillidiopsis*, *Philonotis*, *Pseudosymblepharis*, *Pseudotaxiphyllum*, *Rhynchostegium*, and *Trichosteleum* have one taxon each.

Calymperaceae is indeed a major family in lowland forest areas in Peninsular Malaysia (Damanhuri and Maideen 2001; Damanhuri et al. 2007). In this study area, Fissidentaceae, Hypnaceae, and Neckeraceae are represented by a fairly high number of taxa since the limestone rocks are largely a habitat of choice for the members of these families (Mohamed 1987).

The moss species found in Gunung Senyum Recreational Forest is compared to three other limestone forests in Peninsular Malaysia using Jaccard Coefficient of Similarity. Other limestone forest selected for comparison are Taman Rimba Kenong in Pahang (Damanhuri et al. 2007); Taman Negeri Perlis in Wang Kelian, Perlis (Damanhuri and Maideen 2001) and Gunung Kanthan in Perak (Kiew et al. 2014). Taman Rimba Kenong has the highest number of taxa, 114 taxa, followed by Taman Negeri Perlis in Wang Kelian and Gunung Senyum Recreational Forest with 72 and 66 taxa respectively. Gunung Kanthan recorded the lowest number of taxa, just 25 (Table 3).

**Table 3.** Summary comparing the number of moss taxa in the four areas.

**Locality**	**Families**	**Genera**	**Taxa**
Gunung Senyum	16	32	59 spp. + 2 subsp. + 5 var.
Wang Kelian	18	34	67 spp. + 1 subsp. + 3 var.
Taman Rimba Kenong	19	45	94 spp. + 4 subsp. + 16 var.
Gunung Kanthan	9	14	23 spp. + 2 var.

Taman Negeri Perlis in Wang Kelian exhibited the highest degree of species similarity with Gunung Senyum Recreational Forest, which is 38.8% (Table 4). This is due to both areas consisting of limestone forests. Topographic factors also play an important role in shaping the vegetative patterns that are present in certain areas. Taman Rimba Kenong shows the second highest similarity with Gunung Senyum Recreational Forest, which is 33.6%, meanwhile, Gunung Kanthan show the lowest degree of similarity with this forest which is 19.7%. This may be due to the fact that the number of mosses found in Gunung Kanthan is distinctly low compared to other comparable areas because the plant biodiversity in this location has been under threat from quarrying activity conducted there (Kiew et al. 2014).

**Table 4.** Summary of the level of similarity of moss flora in selected areas.

	**Gunung Senyum**	**Taman Rimba Kenong**	**Wang Kelian**
Taman Rimba Kenong	33.6%		
Wang Kelian	38.8%	36.6%	
Gunung Kanthan	19.7%	15.0%	15.9%

The limestone habitats found in Gunung Senyum Recreational Forest can be divided into five groups based on classification by Chin (1977) and Mohamed (1987). The subdivisions are:

**1. Base of hills.** Species that live in this area include the foothills and the surrounding area. Examples are: *Acanthorrhynchiumpapillatum*, *Acroporiumlamprophyllum*, *Calymperesafzelii*, *C.graeffeanum*, *Fissidenshollianus* and *Vesiculariareticulata*.

**2. Talus slopes.** Species that live in areas which cover the caves at the foot of Gunung Senyum, comprising piles of debris including limestone debris resulted from the weathering process of the rocks, Examples are: *Bryumcoronatum*, *Caduciellamariei*, *Calymperesboulayi* and *C.erosum*.

**3. Gullies and valleys.** This area has plenty of sheltered places and can trap enough water. Examples are: *Fissidensceylonensis*, *Macromitriummiquelii*, and *Neckeropsislepineana*.

**4. Cliffs and near-vertical slopes.** This area provides a unique habitat for mosses because it supports very different vegetation depending on the degree of cliff gradient, presence and absence of soil and humidity levels. Examples are: *Calymperesmoluccense*, *C.taitense*, *Ectropotheciumdealbatum*, *Fissidensoblongifolius*, *Isopterygiumpohliaecarpum* and *Pseudosymblepharisbombayensis*.

**5. Summits.** The summit of Gunung Senyum is an area composed of exposed rocks with only a small land cover. Examples are: *Floribundariafloribunda*, *Hyophilainvoluta*, Isopterygiumalbescensvar.albescens and *Neckeropsislepineana*.

Species found in this study can also be divided into four categories on the basis of their affinity to the limestone habitat (Mohamed 1987)

**1. Exclusives.** Only for species which are solely retricted to the limestone. Examples are: *Pseudosymblepharisbombayensis*, Fissidenscf.hillianus and *F.oblongifolius*.

**2. Preferents.** Occur mainly on limestone (50 to 75% of the time) but also found in non-limestone habitats. Examples are: *Barbulaconsanguinea*, *Bryumcoronatum*, *Calymperestaitense*, *Hyophilainvoluta*, *Neckeropsislepineana*, *Pelekiumvelatum* and *Pinnatellaambigua*.

**3. Indifferents.** Species with no particular preference for either limestone or non-limestone habitats. Examples are: *Calymperesafzelii, C.boulayi*, *C.taitense, Ectropotheciumperminutum*, *Fissidensceylonensis*, *Homaliodendronexiguum* and *Leucophanesoctoblepharioides*.

**4. Casuals.** Non-limestone mosses which are collected on limestone. Example is: *Ectropotheciumdealbatum*.

In this study, corticol is the most dominant way of life for mosses collected in this area with 47 taxa. Lignicol is second with 35 taxa, followed by calcicol or live on limestone with 26 taxa, then the terricol, with about 16 taxa. Rupicol and ramicol recorded the lowest number of taxa with eight and one taxa only. No moss species is found growing on leaves.

The mosses in Gunung Senyum Recreational Forest mostly live as corticol because this habitat provides adequate nutrients, water and exposure to sunlight. In contrast to the limestone rock which has few resources, the growth of mosses here is limited. The land surface is often limited to interstitials and limestone depressions. This factor makes the limestone environment vulnerable and hotter, even the absorption capacity of the soil is limited (Crowther 1987). In addition, the hot environment also accelerates decomposition of humus, thereby reducing the growth of mosses on decomposed materials.

### List of mosses taxa found in Gunung Senyum Recreational Forest, Taman Rimba Kenong, Wang Kelian State Park and Gunung Kanthan

The arrangement of moss taxa are arranged alphabetically. The accepted taxa for Peninsular Malaysia follow Yong et al. (2013) except for *Pelekiumbifarium* (Norhazrina et al. 2017).

**Table:** 

Taxa	G. Senyum	T. R. Kenong	Wang Kelian	G. Kanthan
*Acanthorrhynchiumpapillatum* (Harv.) M. Fleisch.	/	/	/	–
*Acroporiumadspersum* (Hampe) Broth.	–	/	–	–
*Acroporiumjohannis-winkleri* Broth.	–	/	–	–
*Acroporiumlamprophyllum* Mitt.	/	–	–	–
*Acroporiumrufum* (Reinw. & Hornsch.) M. Fleisch.	–	/	–	–
*Aerobryidiumaureonitens* (Hook. *ex* Schwägr.) Broth.	–	/	/	–
*Aerobryidiumcrispifolium* (Broth. & Geh.) M. Fleisch.	–	/	/	–
Aerobryopsis longissima (Dozy & Molk.) M. Fleisch. var. longissima	–	/	–	/
*Arthrocormusschimperi* (Dozy & Molk.) Dozy & Molk.	/	/	/	–
*Barbulaconsanguinea* (Thwaites & Mitt.) A. Jaeger	/	–	/	–
*Bryumapiculatum* Schwägr.	–	/	–	/
*Bryumcoronatum* Schwägr.	/	–	/	/
*Caduciellamariei* (Besch.) Enroth	/	/	/	–
Callicostella papillata (Mont.) Mitt. var. papillata	/	/	–	–
Callicostellapapillata(Mont.)Mitt.var.prabaktiana (Müll. Hal.) Streimann	–	/	–	–
*Calymperesaeruginosum* Hampe *ex* Sande Lac.	–	/	/	–
*Calymperesafzelii* Sw.	/	/	/	–
*Calymperesboulayi* Besch.	/	–	/	/
*Calympereserosum* Müll. Hal.	/	/	/	/
*Calymperesgraeffeanum* Müll. Hal.	/	/	/	–
CalympereslonchophyllumSchwägr.subsp.beccarii (Hampe) M. Menzel	/	/	–	–
Calymperes lonchophyllum Schwägr. subsp. lonchophyllum	/	/	/	–
*Calymperesmoluccense* Schwägr.	/	/	/	–
CalymperesporrectumMitt.var.elatissimum (M. Fleisch.) A. Eddy	–	/	–	–
Calymperes porrectum Mitt. var. porrectum	–	/	–	–
*Calymperesrobinsonii* B.C. Tan & W.D. Reese	–	/	–	–
*Calymperesschmidtii* Broth.	–	–	/	–
*Calymperesserratum* A. Braun *ex* Müll. Hal.	/	/	/	–
*Calymperespallidum* Mitt.	/	–	–	–
*Calymperesstrictifolium* (Mitt.) G. Roth	–	/	–	–
*Calymperestaitense* (Sull.) Mitt.	/	/	/	/
*Calymperestenerum* Müll. Hal.	/	–	/	–
*Chaetomitriumborneense* Mitt.	–	/	–	–
*Chaetomitriumorthorrhynchum* (Dozy & Molk.) Bosch & Sande Lac.	–	/	–	–
*Chaetomitriumpapillifolium* Bosch & Sande Lac.	–	/	/	–
*Circulifoliumexiguum* (Bosch & Sande Lac.) S. Olsson, Enroth & D. Quandt	/	/	/	–
*Circulifoliummicrodendron* (Mont.) S. Olsson, Enroth & D. Quandt	–	/	/	–
*Cryptogoniumphyllogonioides* (Sull.) Isov.	–	–	/	–
*Desmothecaapiculata* (Dozy & Molk.) Lindb.	–	/	–	–
*Dimorphocladonborneense* Mitt.	–	/	–	–
*Diphysciummucronifolium* Mitt.	–	/	–	–
*Ectropothecielladistichophylla* (Hampe *ex* Dozy & Molk.) M. Fleisch.	–	/	–	–
*Ectropotheciumbuitenzorgii* (Bél.) Mitt.	–	/	/	–
*Ectropotheciumdealbatum* (Reinw. & Hornsch.) A. Jaeger	/	–	–	–
*Ectropotheciumeleganti-pinnatum* (Müll. Hal.) A. Jaeger	–	/	–	–
*Ectropotheciumichnotocladum* (Müll. Hal.) A. Jaeger	–	/	–	–
*Ectropotheciummonumentorum* (Duby) A. Jaeger	–	/	–	/
*Ectropotheciumperminutum* Broth. *ex* E.B. Bartram	/	/	/	–
*Ectropotheciumzollingeri* (Müll. Hal.) A. Jaeger	–	–	–	/
*Ephemeropsistjibodensis* K.I. Goebel	–	/	–	–
*Erythrodontiumjulaceum* (Hook. *ex* Schwägr.) Paris	–	/	–	–
*Exostratumasperum* (Mitt.) L.T. Ellis	–	/	–	–
*Exostratumblumii* (Nees *ex* Hampe) L.T. Ellis	/	/	/	–
*Fissidensbogoriensis* M. Fleisch.	–	–	/	–
FissidensbryoidesHedw.var.ramossissimus Thér.	/	–	–	–
*Fissidensceylonensis* Dozy & Molk.	/	/	/	/
Fissidens crassinervis Sande Lac. var. crassinervis	/	/	–	–
FissidenscrenulatusMitt.var.elmeri (Broth.) Z. Iwats. & Tad. Suzuki	/	/	–	–
Fissidens crispulus Brid. var. crispulus	–	/	/	/
FissidenscrispulusBrid.var.robinsonii (Broth.) Z. Iwats. & Z.H. Li	–	/	/	–
*Fissidensflaccidus* Mitt.	–	–	/	–
*Fissidensguangdongensis* Z. Iwats. & Z.H. Li	–	/	–	–
*Fissidenshollianus* Dozy & Molk.	/	/	/	/
*Fissidensjavanicus* Dozy & Molk.	–	/	/	–
*Fissidensoblongifolius* Hook. f. & Wilson	/	/	/	/
*Fissidenspellucidus* Hornsch.	/	/	/	–
*Fissidens* sp.	–	–	–	/
*Fissidens* sp. A	/	–	–	–
Fissidens cf. hillianus	/	–	–	–
*Fissidenszollingeri* Mont.	/	–	/	–
*Floribundariafloribunda* (Dozy & Molk.) M. Fleisch.	/	/	–	/
*Groutiellatomentosa* (Hornsch.) Wijk & Margad.	–	–	/	–
*Himantocladiumcyclophyllum* (Müll. Hal.) M. Fleisch.	–	/	–	–
*Himantocladiumplumula* (Nees) M. Fleisch	–	–	/	–
*Hymenostyliumrecurvirostrum* (Hedw.) Dixon	–	/	–	–
*Hyophilainvoluta* (Hook.) A. Jaeger	/	/	/	–
*Hyophilajavanica* (Nees & Blume) Brid.	–	–	/	–
*Hyophilarosea* R.S. Williams	–	–	/	–
Isopterygium albescens (Hook.) A. Jaeger var. albescens	/	/	/	–
Leucobryum aduncum Dozy & Molk. var. aduncum	–	/	/	–
*Leucobryumcandidum* (Brid. *ex* P. Beauv.) Wilson	–	/	–	–
*Leucobryumjavense* (Brid.) Mitt.	–	/	–	–
*Leucobryumsanctum* (Nees *ex* Schwägr.) Hampe var. sanctum	–	/	–	–
*Leucolomawalkeri* Broth.	–	–	/	–
*Leucomiumstrumosum* (Hornsch.) Mitt.	/	–	–	–
*Leucophanesangustifolium* Renault & Cardot	/	/	/	–
Leucophanes candidum (Schwägr.) Lindb. var. candidum	–	/	–	–
*Leucophanesglaucum* (Schwägr.) Mitt.	–	/	/	–
*Leucophanesoctoblepharioides* Brid.	/	/	/	–
*Macromitriumangustifolium* Dozy & Molk.	–	/	/	–
*Macromitriumblumei* Nees *ex* Schwägr. var. zolligeri (Mitt. *ex* Bosch & Sande Lac.) S.L. Guo, B.C. Tan & Virtanen	–	/	–	–
*Macromitriumfalcatulum* Müll. Hal.	–	/	/	–
*Macromitriumfuscescens* Schwägr.	/	/	–	–
Meiothecium microcarpum (Harv.) Mitt. var. microcarpum	/	–	–	–
*Meteoriumpolytrichum* Dozy & Molk.	–	/	/	–
*Mitthyridiumconstrictum* (Sull.) H. Rob.	–	/	–	–
Mitthyridiumfasciculatum(Hook. & Grev.)H. Rob.subsp.cardotii (M. Fleisch.) B.C. Tan & L.T. Ellis	–	/	–	–
Mitthyridium fasciculatum (Hook. & Grev.) H. Rob. subsp. fasciculatum	–	/	–	–
*Mitthyridiumflavum* (Müll. Hal.) H. Rob.	/	/	/	–
*Mitthyridiumjungquilianum* (Mitt.) H. Rob.	–	/	–	–
*Mitthyridiumrepens* (Harv.) H. Rob.	/	/	–	–
*Mitthyridiumundulatum* (Dozy & Molk.) H. Rob.	/	/	/	–
Mitthyridium wallisii (Müll. Hal.) H. Rob. var. wallisii	–	/	–	–
*Neckeropsisandamana* (Müll. Hal.) M. Fleisch.	–	–	/	–
*Neckeropsisfleischeri* (Dixon) A. Touw	–	/	–	–
*Neckeropsisgracilenta* (Bosch & Sande Lac.) M. Fleisch.	/	/	–	/
*Neckeropsislepineana* (Mont.) M. Fleisch.	/	/	/	/
*Octoblepharumalbidum* Hedw.	–	–	/	–
*Oedicladiumpseudorufescens* (Hampe) B.C. Tan & Mohamed	–	/	/	–
*Orthodontiuminfractum* Dozy & Molk.	–	–	/	–
*Papillidiopsiscomplanata* (Dixon) W.R. Buck & B.C. Tan	/	/	–	–
*Papillidiopsismalesiana* W.R. Buck & B.C. Tan	–	/	–	–
*Pelekiumbifarium* (Bosch & Sande Lac.) M. Fleisch.	/	/	/	–
*Pelekiumgratum* (P. Beauv.) A. Touw	–	–	–	/
*Pelekiumvelatum* Mitt.	/	/	/	/
*Philonotishastata* (Duby) Wijk & Marg.	/	–	–	–
*Pinnatellaalopecuroides* (Hook.) M. Fleisch.	–	–	/	–
*Pinnatellaambigua* (Bosch & Sande Lac.) M. Fleisch.	/	/	/	–
*Pinnatellacalcutensis* M. Fleisch.	–	–	/	–
*Pinnatellakuehliana* (Bosch & Sande Lac.) M. Fleisch.	–	/	–	/
*Pinnatellamucronata* (Bosch & Sande Lac.) M. Fleisch.	/	/	/	/
*Pogonatumpiliferum* (Dozy & Molk.) Lindb.	–	/	–	–
*Pseudosymblepharisbombayensis* (Müll. Hal.) P. Sollman	/	/	/	/
*Pseudotaxiphyllumpohliaecarpum* (Sull. & Lesq.) Z. Iwats.	/	–	–	–
*Rhynchostegiumcelebicum* (Sande Lac.) A. Jaeger	–	/	–	–
*Rhynchostegiumjavanicum* (Bél.) Besch.	/	–	–	–
*Stereodontopsisexcavata* (Broth.) Ando	–	/	–	–
*Stereophyllumtavoyense* (Hook.) A. Jaeger	–	–	/	–
*Syrrhopodonalbo-vaginatus* Schwägr.	/	/	/	–
*Syrrhopodonaristifolius* Mitt.	–	/	–	–
*Syrrhopodonconfertus* Sande Lac.	–	/	–	–
*Syrrhopodoncroceus* Mitt.	–	/	–	–
*Syrrhopodoninvolutus* Schwägr.	–	/	–	–
*Syrrhopodonloreus* (Sande Lac.) W.D. Reese	–	/	–	–
*Syrrhopodonmuelleri* (Dozy & Molk.) Sande Lac.	/	/	/	–
Syrrhopodon prolifer Schwägr. var. prolifer	–	/	–	–
Syrrhopodon spiculosus Hook. & Grev. var. spiculosus	–	/	/	–
*Syrrhopodontrachyphyllus* Mont.	–	/	–	–
*Syrrhopodontristichus* Nees *ex* Schwägr.	–	/	–	–
*Taxiphyllumtaxirameum* (Mitt.) M. Fleisch.	–	/	–	/
*Taxitheliumbinsteadii* Broth. & Dixon	/	–	–	–
*Taxitheliuminstratum* (Brid.) Broth.	/	/	/	–
*Taxitheliumisocladum* (Bosch & Sande Lac.) Renauld & Cardot	/	/	–	–
*Taxitheliumnepalense* (Schwägr.) Broth.	/	–	/	/
*Taxitheliumkerianum* (Broth.) Broth.	/	–	–	–
*Thuidiumplumulosum* (Dozy & Molk.) Dozy & Molk.	–	/	/	–
Thuidium pristocalyx (Müll. Hal.) A. Jaeger var. pristocalyx	–	/	–	–
*Trichosteleumboschii* (Dozy & Molk.) A. Jaeger	/	–	–	–
*Trichostomumbrachydontium* Bruch	–	–	–	/
Trismegistia lancifolia (Harv.) Broth. var. lancifolia	–	/	–	–
*Vesiculariadubyana* (Müll. Hal.) Broth.	/	–	–	–
*Vesiculariamiquelii* (Sande Lac.) M. Fleisch.	/	/	–	–
*Vesiculariamontagnei* (Schimp.) Broth.	/	/	–	/
*Vesiculariareticulata* (Dozy & Molk.) Broth.	/	/	–	–

## Conclusion

Gunung Senyum Recreational Forest with its two unique and pristine limestone outcrops is suitable for exploration and study as this forested limestone area is inhabited by interesting flora and fauna. It is hoped that in future, the composition of moss flora in these limestone outcrops, Gunung Senyum and Gunung Jebak Puyuh, will be studied more deeply. Specimens from the steep cliffs in this area should be collected regularly to assess the true diversity of mosses in this unique limestone hill.

## Appendix 1. Moss checklist of Gunung Senyum Recreational Forest, Pahang

The arrangement of moss taxa follows Goffinet and Buck (2018), and families as well as the genera under each family and species under each genus are arranged alphabetically. The accepted taxa for Peninsular Malaysia follow Yong et al. (2013) except for *Pelekiumbifarium* (Norhazrina et al. 2017). New additions to the bryoflora of Peninsular Malaysia and the state of Pahang are indicated by ‘**’ and ‘*’ respectively.

BARTRAMIACEAE

***Philonotis* Brid.**

*P.hastata* (Duby) Wijk & Margad. – H. Aznani & A. Damanhuri 20, 149 & 160. On exposed roots and on limestone.

BRACHYTHECIACEAE

***Rhynchostegium* Bruch & Schimp.**

*R.javanicum* (Bél.) Besch. – H. Aznani & A. Damanhuri 339, 347 & 422. On base of trees and on limestone.

BRYACEAE

***Bryum* Hedw.**

*B.coronatum* Schwägr. – H. Aznani & A. Damanhuri 24, 136, 154, 153, 157, 158, 163, 167, 168, 176, 178, 179, 188, 205, 206, 208, 210, 212, 219, 220, 221, 225, 228, 234, 236, 237, 238, 241, 243, 248, 263 & 264. On rotten logs, on soil, on tree trunks and on limestone.

CALYMPERACEAE

***Arthrocormus* Dozy & Molk.**

*A.schimperi* (Dozy & Molk.) Dozy & Molk. – H. Aznani & A. Damanhuri 458, 514 dan 522. On buttresses and base of trees.

***Calymperes* Sw. *ex* F. Weber**

*C.afzelii* Sw. – H. Aznani & A. Damanhuri 271, 278, 280, 284, 315, 326, 348, 361, 397, 398, 399, 415, 436, 477, 481 & 502. On base of trees and on buttresses.

*C.boulayi* Besch. – H. Aznani & A. Damanhuri 11, 12, 204, 218, 229 & 251. On limestone.

*C.erosum* Müll. Hal. – H. Aznani & A. Damanhuri 56a. On rotten log.

*C.graeffeanum* Müll. Hal. – H. Aznani & A. Damanhuri 405. On rotten log.

C.lonchophyllumsubsp.beccarii (Hampe) M. Menzel – H. Aznani & A. Damanhuri 350. On exposed roots.

C.lonchophyllumsubsp.lonchophyllum Schwägr. – H. Aznani & A. Damanhuri 287, 302, 311, 312, 330, 368 & 429. On exposed roots and on base of trees.

*C.moluccense* Schwägr. – H. Aznani & A. Damanhuri 116a. On tree trunk.

***C.pallidum* Mitt. - H. Aznani & A. Damanhuri 56a. On burnt wood.

*C.serratum* A. Braun *ex* Müll. Hal. – H. Aznani & A. Damanhuri 294. On rotten log.

*C.taitense* (Sull.) Mitt. – H. Aznani & A. Damanhuri 91, 109, 117, 123, 133, 169, 286, 346, 370, 438 & 451. On exposed roots, on limestone and on bark of liana.

*C.tenerum* Müll. Hal. – H. Aznani & A. Damanhuri 387. On rotten log.

***Exostratum* L.T. Ellis**

*E.blumii* (Nees *ex* Hampe) L.T. Ellis – H. Aznani & A. Damanhuri 324, 393, 394, 400 & 424. On rotten log.

***Leucophanes* Brid.**

*L.angustifolium* Renauld & Cardot – H. Aznani & A. Damanhuri 316, 337, 376, 369, 400, 457, 490 & 498. On buttresses, on base of tree and on rotten log.

*L.octoblepharioides* Brid. – H. Aznani & A. Damanhuri 270, 284, 323, 347, 378, 395, 404, 443, 470, 487, 495, 512, 535 & 537. On base of trees, on roots of fern, on limestone and on rotten log.

***Mitthyridium* H. Rob.**

*M.flavum* (Müll. Hal.) H. Rob. – H. Aznani & A. Damanhuri 274, 309, 345, 390, 410, 439, 448, 475, 499, 505, 534 & 558. On base of trees and on rotten log.

*M.repens* (Harv. *in* Hook.) H. Rob. – H. Aznani & A. Damanhuri 585. On tree trunk.

*M.undulatum* (Dozy & Molk.) H. Rob. – H. Aznani & A. Damanhuri 478. On base of tree.

***Syrrhopodon* Schwägr.**

*S.albo-vaginatus* Schwägr. – H. Aznani & A. Damanhuri 214, 294, 298, 314, 329, 372, 403, 507, 534, 545, 546, 578 & 579. On buttresses, on tree trunks and on rotten log.

*S.muelleri* (Dozy & Molk.) Sande Lac. – H. Aznani & A. Damanhuri 513 dan 526. On base of trees.

FISSIDENTACEAE

***Fissidens* Hedw.**

F.bryoidesvar.ramosissimus Thér. – H. Aznani & A. Damanhuri 396. On tree trunk.

*F.ceylonensis* Dozy & Molk. – H. Aznani & A. Damanhuri 1, 18, 55, 64a, 65, 80, 108, 115, 124, 125, 130, 140, 142, 143, 146, 148, 151, 152, 156, 158, 161, 166, 170, 201 & 560. On limestone, on soil, on rock in the crevice of trees and on buttress.

*F.crassinervis* Sande Lac. – H. Aznani & A. Damanhuri 81, 289, 442, 426, 570, 577, 580, 583 & 584. On soil, on rocks and on soil between exposed roots.

F.crenulatusvar.elmeri (Broth.) Z. Iwats. & Tad. Suzuki – H. Aznani & A. Damanhuri 519. On exposed roots.

F.cf.hillianus H.A. Mill. & D.R. Sm. – H. Aznani & A. Damanhuri 35a, 88, 98, 99, 103 & 147. On limestone.

*F.hollianus* Dozy & Molk. – H. Aznani & A. Damanhuri 31, 48, 49, 61, 78, 93, 97, 121, 141, 126, 265, 284, 287, 290, 318, 375, 382, 383, 388, 396, 406, 414, 416, 454, 464, 491, 499, 518, 557, 571 & 575. On roots, on base and tree trunk, on rotten log and on burnt wood.

*F.oblongifolius* Hook. f. & Wilson – H. Aznani & A. Damanhuri 92, 107, 110, 112, 114, 122, 127, 131, 134, 139, 155, 180, 231 & 239. On soil, on tree trunks and on limestone.

*F.pellucidus* Hornsch. – H. Aznani & A. Damanhuri 425 & 510. On soil and at base of tree.

*F.zollingeri* Mont. – H. Aznani & A. Damanhuri 6, 9, 13a, 14, 42, 360 & 504. On rocks, on termite hill and on soil.

HYPNACEAE

***Ectropothecium* Mitt.**

*E.dealbatum* (Reinw. & Hornsch.) A. Jaeger – H. Aznani & A. Damanhuri 152. On limestone.

*E.perminutum* Broth. *ex* E.B. Bartram – H. Aznani & A. Damanhuri 8 & 56b. On rotten log.

***Vesicularia* (Müll. Hal.) Müll. Hal.**

*V.dubyana* (Müll. Hal.) Broth. – H. Aznani & A. Damanhuri 4, 19, 64, 295, 351, 384, 392a, 402, 431, 445, 456 & 459. On roots and base of tree, on soil and on rotten logs.

*V.miquelii* (Sande Lac.) M. Fleisch. – H. Aznani & A. Damanhuri 298, 444, 458 & 545. On buttresses and on rotten logs.

*V.montagnei* (Schimp.) Broth. – H. Aznani & A. Damanhuri 5, 44, 127, 317, 342, 366, 426, 430, 449, 461, 533 & 566. On soil, on tree trunks and on rotten logs.

*V.reticulata* (Dozy & Molk.) Broth. – H. Aznani & A. Damanhuri 335, 343, 362, 472, 497a & 509. On rotten log and on base of trees.

LEUCOMIACEAE

***Leucomium* Mitt.**

*L.strumosum* (Hornsch.) Mitt. – H. Aznani & A. Damanhuri 423. On rotten log.

METEORIACEAE

***Floribundaria* M. Fleisch.**

*F.floribunda* (Dozy & Molk.) M. Fleisch. – H. Aznani & A. Damanhuri 245. On bark of liana.

NECKERACEAE

***Caduciella* Enroth**

*C.mariei* (Besch.) Enroth – H. Aznani & A. Damanhuri 40, 353, 355, 356, 373, 418, 446, 452, 471, 473, 480, 486, 492, 520, 528a, 548 & 555. On exposed roots and on base of trees.

***Circulifolium* S. Olsson, Enroth & D. Quandt**

*C.exiguum* (Bosch & Sande Lac.) S. Olsson, Enroth & D. Quandt – H. Aznani & A. Damanhuri 17, 29, 36, 39, 45, 50, 72, 89, 94, 100, 102, 241, 279, 281, 291, 296, 313, 332, 352, 355a, 380, 392, 455, 462, 469, 511, 528 & 551. On limestone, on exposed roots, on soil, on rotten logs and on base of trees.

***Neckeropsis* Reichardt**

*N.gracilenta* (Bosch & Sande Lac.) M. Fleisch. – H. Aznani & A. Damanhuri 250, 253, 255, 258, 306, 340, 401, 440, 503, 509, 525, 529, 530, 543 & 556. On base of trees.

*N.lepineana* (Mont.) M. Fleisch. – H. Aznani & A. Damanhuri 116, 137, 164, 169, 177, 196, 215, 247 & 242. On limestone and on the edge of boulders.

***Pinnatella* M. Fleisch.**

*P.ambigua* (Bosch & Sande Lac.) M. Fleisch. – H. Aznani & A. Damanhuri 291a, 340 & 358a. On limestone and on base of trees.

*P.mucronata* (Bosch & Sande Lac.) M. Fleisch. – H. Aznani & A. Damanhuri 377, 402, 539, 540a & 552. On base of trees.

ORTHOTRICHACEAE

***Macromitrium* Brid.**

*M.fuscescens* Schwägr. – H. Aznani & A. Damanhuri 209. On rotten log.

PILOTRICHACEAE

***Callicostella* (Müll. Hal.) Mitt.**

C.papillata(Mont.)Mitt.var.papillata – H. Aznani & A. Damanhuri 283, 329, 393 & 566a. On rotten log, on buttresses and on tree trunks.

PLAGIOTHECIACEAE

***Pseudotaxiphyllum* Z. Iwats.**

*P.pohliaecarpum* (Sull. & Lesq.) Z. Iwats. – H. Aznani & A. Damanhuri 34, 67, 90 103 138, 195, 241a, 254, 324, 357, 406 & 497a (as *Isopterygiumminutirameum*). On exposed roots, on soil, on limestone, on tree trunks and on rotten logs.

POTTIACEAE

***Barbula* Hedw.**

*B.consanguinea* (Thwaites & Mitt.) A. Jaeger – H. Aznani & A. Damanhuri 190. On limestone.

***Hyophila* Brid.**

*H.involuta* (Hook.) A. Jaeger – H. Aznani & A. Damanhuri 21, 41, 106, 244 & 252. On rocks, on roots of fern and on limestone.

***Pseudosymblepharis* Broth**

*P.bombayensis* (Müll. Hal.) P. Sollman – H. Aznani & A. Damanhuri 101, 105, 171, 118, 142, 145, 153a, 155, 159, 162, 172, 173, 174, 175, 181, 182, 183, 185, 187, 189, 193, 199, 203, 207, 211, 213, 214, 222, 224, 225, 227, 235, 240 & 249. On limestone.

PYLAISIADELPHACEAE

***Isopterygium* Mitt.**

I.albescens(Hook.)A. Jaegervar.albescens – H. Aznani & A. Damanhuri 8, 10, 15, 22, 35, 119, 121a, 129, 275, 292, 300, 305, 450, 506, 508, 516, 523, 527, 532 & 539. On rotten log, on soil, on exposed roots, on base of trees, on bark of lianas, on buttresses and on limestone.

***Taxithelium* Spruce *ex* Mitt.**

**T.binsteadii* Broth. & Dixon – H. Aznani & A. Damanhuri 588. On tree trunk.

*T.instratum* (Brid.) Broth. – H. Aznani & A. Damanhuri 126, 127, 307, 312, 320, 333, 336, 359, 391, 413, 433, 437, 439, 466, 467 & 501. On rotten logs, on soil, on exposed roots, on limestone and on bark of lianas.

*T.isocladum* (Bosch & Sande Lac.) Renauld & Cardot – H. Aznani & A. Damanhuri 288 & 476. On exposed roots.

*T.kerianum* (Broth.) Broth. – H. Aznani & A. Damanhuri 275a, 283, 309, 321, 381, 474 & 573. On base of trees.

*T.nepalense* (Schwägr.) Broth. – H. Aznani & A. Damanhuri 46, 53, 73, 269, 273, 299, 304, 305, 402 & 533. On rotten log, on exposed roots, on stumps of tree inside the lake and on wood.

SEMATOPHYLLACEAE

***Acanthorrhynchium* M. Fleisch.**

*A.papillatum* (Harv.) M. Fleisch. – H. Aznani & A. Damanhuri 444a & 458. On buttresses.

***Acroporium* Mitt.**

*A.lamprophyllum* Mitt. – H. Aznani & A. Damanhuri 586 & 587. On rotten logs.

***Meiothecium* Mitt.**

*M.microcarpum* (Harv.) Mitt. var. microcarpum – H. Aznani & A. Damanhuri 30, 217, 362 & 524. On limestone, on tree trunks and on rotten logs.

***Papillidiopsis* (Broth.) W.R. Buck & B.C. Tan**

*P.complanata* (Dixon) W.R. Buck & B.C. Tan – H. Aznani & A. Damanhuri 386, 493, 531, 549, 561, 572, 574, 582 & 586. On rotten logs, on tree trunks and branches.

***Trichosteleum* Mitt.**

*T.boschii* (Dozy & Molk.) A. Jaeger – H. Aznani & A. Damanhuri 303, 314a, 485, 544 & 581. On soil and rotten logs.

THUIDIACEAE

***Pelekium* Mitt.**

*P.bifarium* (Bosch & Sande Lac.) M. Fleisch. - H. Aznani & A. Damanhuri 3, 23, 29, 32, 38, 44, 47, 52, 55, 62, 72, 77, 87, 88, 98, 128, 300, 349 & 553 (as *Aequatoriellabifaria*). On limestone, on rotten log, on soil and tree trunks.

*P.velatum* Mitt. - H. Aznani & A. Damanhuri 2, 4, 5, 7, 8, 13, 19, 26, 33, 43, 51, 54, 57, 61, 63, 64, 66, 70, 71, 77, 104, 132, 269, 272, 296, 297, 301, 308, 322, 328, 325, 326, 334, 341, 344, 347a, 358, 359, 363, 364, 374, 385, 389, 391, 392, 402, 406, 407, 408, 411, 412, 417, 419, 428, 432, 435, 437, 441, 456, 463, 468, 472, 479, 480, 483, 488, 494, 500, 536, 550, 554, 559, 563, 564, 565, 567 & 569. On limestone, on rocks, on soil, on tree trunks, on rotten logs, on exposed roots and on base of trees.
